# Hypereosinophilia with a pinch of salt and pepper

**DOI:** 10.1002/jha2.255

**Published:** 2021-06-27

**Authors:** Angelantonio Vitucci, Francesco Tarantini, Giuseppe Ingravallo, Giorgina Specchia, Pellegrino Musto, Francesco Albano

**Affiliations:** ^1^ Department of Emergency and Organ Transplantation (D.E.T.O.), Hematology Section University of Bari ‘Aldo Moro’ Bari Italy; ^2^ Department of Emergency and Organ Transplantation (D.E.T.O.), Pathology Section University of Bari, ‘Aldo Moro’ Bari Italy; ^3^ School of Medicine University of Bari ‘Aldo Moro’ Bari Italy


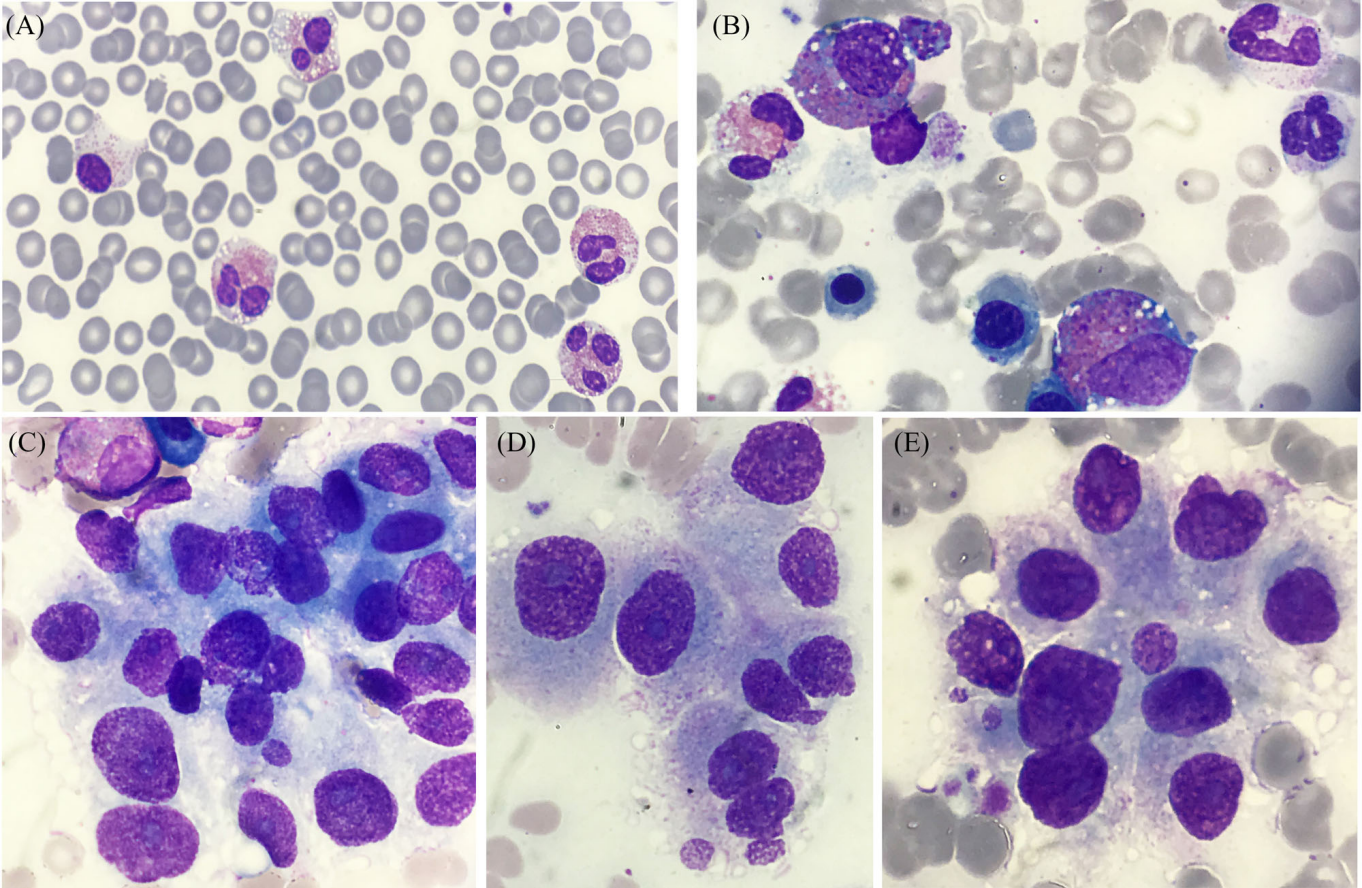


A 49‐year‐old man with a recent medical history of metastatic medullary thyroid carcinoma (stage IVC, spreading to the bone and liver), in palliative treatment, was referred to our centre for hypereosinophilia. His blood count showed: white cell count 58.10 × 10^9^/L, haemoglobin concentration 137 g/L, and platelet count 98 × 10^9^/L. A marked eosinophilia (33 × 10^9^/L) was confirmed. A blood film showed eosinophils dysplastic changes including vacuolation, hypogranularity, hypolobation, and hyperlobation (image A, ×100 magnification). Immunophenotyping did not show evidence of lymphocytes with aberrant T antigens expression. Molecular analysis resulted negative for the *BCR‐ABL1, FIP1L1‐PDGFRA* rearrangement, and *JAK2* gene mutation. A bone marrow aspirate showed around 15% cells belonging to the eosinophil lineage; eosinophilic precursors showed abnormally large, coarse, purple‐violet granules and a basophil cytoplasm (image B, ×100 magnification). Moreover, there were also round‐to‐oval cells arranged in small clumps, whose nuclei exhibited a chromatin granularity (salt and pepper pattern) and prominent nucleoli, separated by an amorphous substance (images C and D, ×100 magnification). These cells sometimes appeared also to be arranged as in microfollicles (image E, ×100 magnification). Bone marrow histopathology revealed the infiltration of a metastatic medullary thyroid carcinoma.

Medullary thyroid carcinoma represents 1%–2% of thyroid carcinomas; one of its peculiar characteristics is a nuclear pleomorphism of cells nuclei and a neuroendocrine‐type salt and pepper chromatin. Hypereosinophilia in this patient was a paraneoplastic sign of a malignant process. The eosinophils abnormalities described in our case can be seen in both reactive and neoplastic eosinophilia. Although eosinophilia is frequently observed in many types of cancers as an expression of a reactive phenomenon, its association with medullary thyroid carcinoma is rare.

## CONFLICT OF INTEREST

The authors declare that there is no relevant conflict of interest.

## AUTHOR CONTRIBUTIONS

Francesco Albano and Angelantonio Vitucci performed morphological analyses. Giuseppe Ingravallo performed bone marrow trephine biopsy analysis. Francesco Tarantini provided clinical data. Francesco Albano, Giorgina Specchia and Pellegrino Musto wrote the manuscript, which was approved by all authors.

